# Dataset on gene expression profiling of multiple murine hair follicle populations

**DOI:** 10.1016/j.dib.2016.08.063

**Published:** 2016-09-05

**Authors:** Anders Patrik Gunnarsson, Rikke Christensen, Jian Li, Uffe Birk Jensen

**Affiliations:** a Department of Clinical Genetics, Aarhus University Hospital, 8200 Aarhus, Denmark; b Institute of Clinical Medicine, Aarhus University, 8000 Aarhus, Denmark; c The Key Laboratory of Developmental Genes and Human Disease, Ministry of Education, Institute of Life Sciences, Southeast University, Nanjing, China; d Department of Biomedicine, Aarhus University, 8000 Aarhus, Denmark

**Keywords:** Epidermal keratinocytes, Stem cells

## Abstract

The murine hair follicle contains several different keratinocyte progenitor populations within its compartments. By using antibodies against CD34, Itgα6, Sca-1 and Plet-1, we have isolated eight populations and compared their Krt10 and Krt14 expressions using fluorescence microscopy. This improved panel was used in our associated article doi:10.1016/j.scr.2016.06.002 (A.P. Gunnarsson, R. Christensen, J. Li, U.B. Jensen, 2016) [1] and the present dataset describes the basic controls for the FACS. We also used imaging flow cytometry to visualize the identified populations as control. A more detailed analysis of the global gene expression profiling is presented, focusing on the pilosebaceous unit. Murine whole-mounts were stained for heat shock protein Hspa2, which is exclusively expressed by keratinocytes with low or no expression of the four selection markers (*IRK*). Whole-mount labeling was also conducted to visualize Krt79 and Plet-1 coexpression within the hair follicle and quantification on the distribution of Krt79 positive keratinocytes is presented.

**Specifications Table**

**Table:** 

Subject area	*Biology.*
More specific subject area	*Epidermal stem cells.*
Type of data	*Images (fluorescence microscopy), diagrams.*
How data was acquired	*Images were acquired using confocal LSM 710 and Leica Letiz DMRB microscopes. Venn diagrams were generated using GeneSpring GX (Agilent) from global microarray gene expression profiling.*
Data format	*Raw, merged, filtered and analyzed.*
Experimental factors	*Keratinocytes were isolated from murine dorsal skin after trypsinization for 16 h at* 4 °C*. Murine tail whole-mounts were obtained by keeping the skin in PBS with 5 mM EDTA for 5 h and* 37 °C.
Experimental features	*Murine tail whole-mounts and isolated populations were stained and visualized using fluorescence microscopy. Differently expressed genes were obtained from microarray data using GeneSpring GX software and ANOVA with Benjamin-Hochberg multiple correction (p-value<0.01).*
Data source location	*AROS Biotechnology (Skejby, Denmark).*
	*Aarhus University (Aarhus Denmark).*
Data accessibility	*Data is within this article.*

**Value of the data**

• To give further insight on how the murine hair follicle keratinocytes can be categorized into different populations.
• To deepen the knowledge on how the populations relate to each other by their gene expression profiles.
• To understand where the different hair follicle populations are located.

## 1 Data

**Fig. 1:**
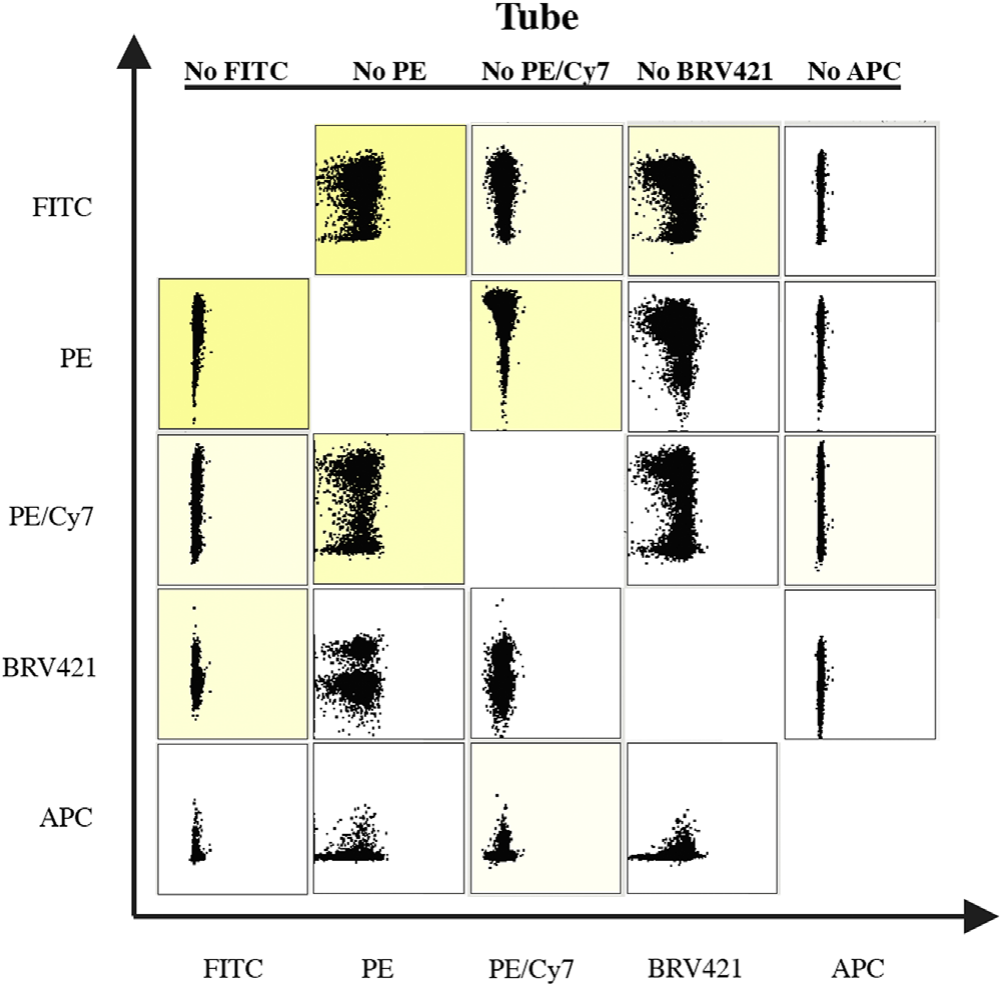
Fluorescence minus one (FMO) assay ensures that no false positive signaling are present. Summarizing chart of multiple flow cytometry dot plot-profiles. Each dot represents one cell that are plotted on its staining intensity of the X- and Y-axis fluorochromes. In five tubes, freshly isolated murine keratinocytes were stained with the antibodies Plet-1-APC, Sca-1-PE/Cy7, Itgα6-PE, CD34-BRV421 and Krt14-FITC. However, each tube only contained four of the five antibodies, excluding one (No FITCH, No PE, No PE/Cy7, No BRV421 and No APC). For each of the excluded antibodies, the flow cytometer will detect any false positive signal that are caused by spectral overlaps from the other fluorochrome emission signals.

**Fig. 2:**
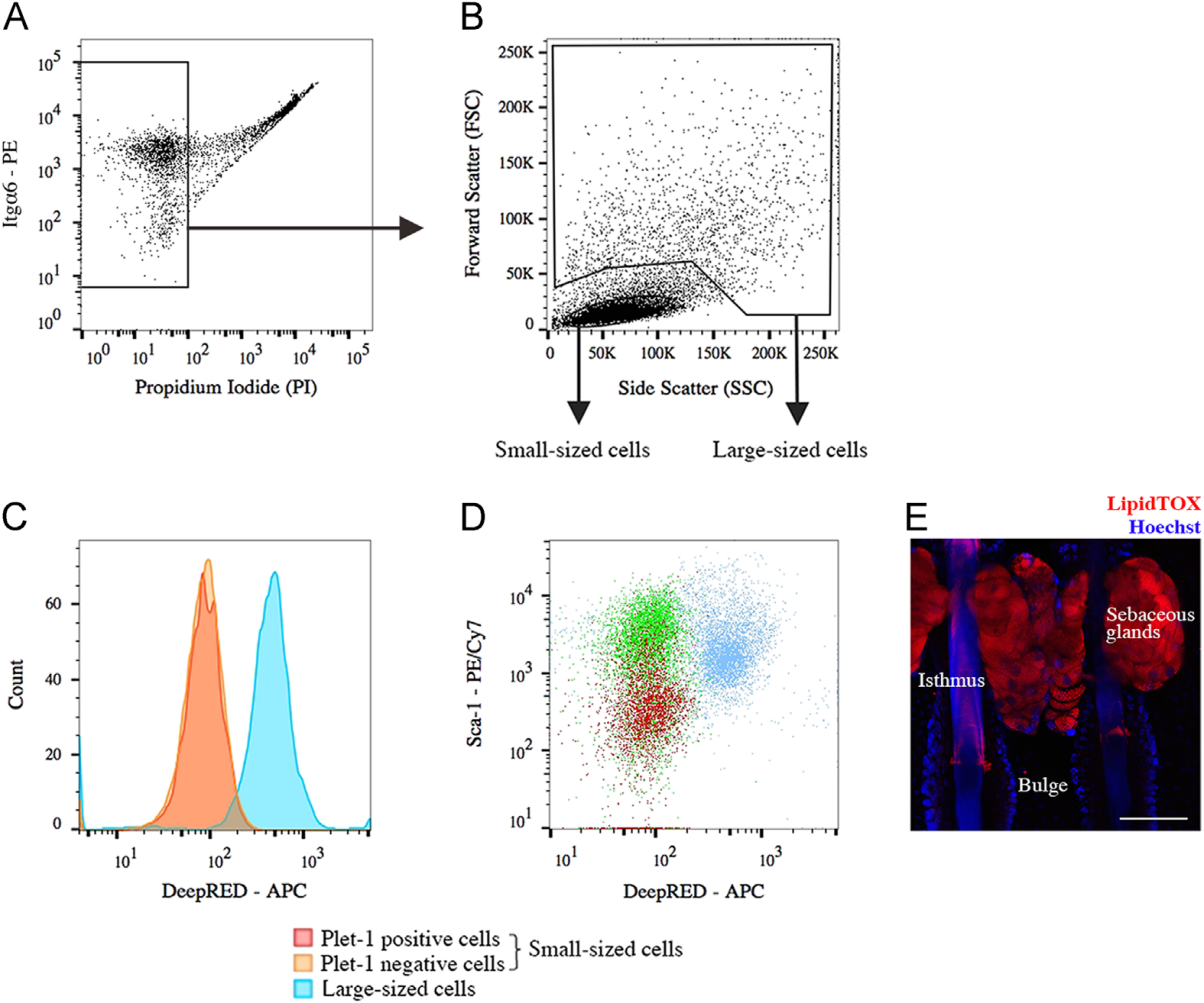
Using flow cytometry, the keratinocytes are first gated on viability and small size for subsequent isolation of the different epidermal populations. To verify that these were not differentiated cells of the sebaceous glands, keratinocyte cell suspension from dorsal skin was stained against Plet-1, Sca-1, Itgα6 and CD34 together with LipidTOX DeepRED that detects lipid droplets. (A) Cells are first selected for viability (negative for Propidium iodide). (B) Live cells are gated into small-sized (low forward- and side scatter) or large-sized (high forward- and side scatter). (C) Histogram illustrating DeepRED staining intensities of the large-sized cells and the small-sized cells, which were separated into Plet-1 positive and Plet-1 negative keratinocytes. (D) Dot plot of Plet-1 positive, Plet-1 negative and large-sized cells based on their Sca-1 and DeepRED intensities. (E) Confocal section view of two murine tail whole-mounts stained with LipidTOX DeepRED (red) and Hoechst (blue). Scale bar: 50 μm.(For interpretation of the references to color in this figure legend, the reader is referred to the web version of this article).

**Fig. 3:**
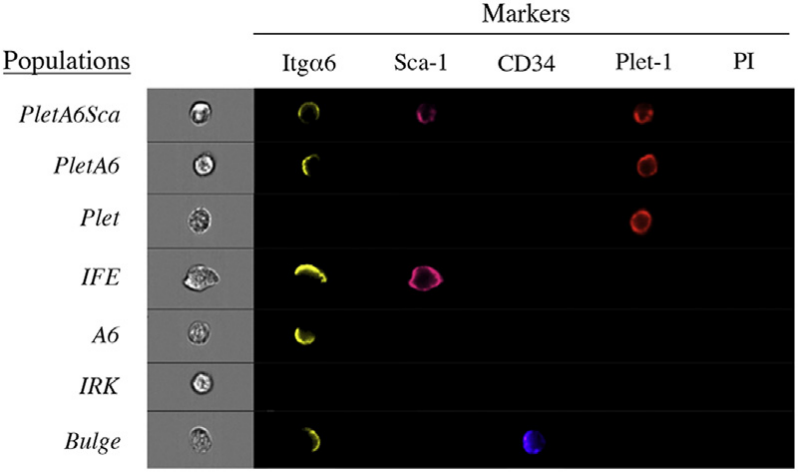
Single cell imaging of the different keratinocyte populations. Freshly isolated murine dorsal keratinocytes were stained with surface-antibodies against Sca-1, CD34, Itgα6 and Plet-1, after which single cell images were acquired using ImageStream flow cytometer. Propidium iodide (PI) was used to exclude dead cells. Polarized Itgα6 staining can be visualized on the cell surface, representing the part of the keratinocytes that is connected to the basal layer [2], [3]. In accordance to the flow cytometry dot plots (Fig. 2D) The population Plet-1 negative population *IFE* generally shows stronger Sca-1 staining than the Plet-1 positive population *PletA6Sca*. In addition, Plet-1 staining were more intense in the *Plet* population compared to *PletA6* and *PletA6Sca*. A polarized cell-surface localization of the CD34 protein is seen at the at the opposite side of Itgα6, which can be visualized in *Bulge*.

**Fig. 4:**
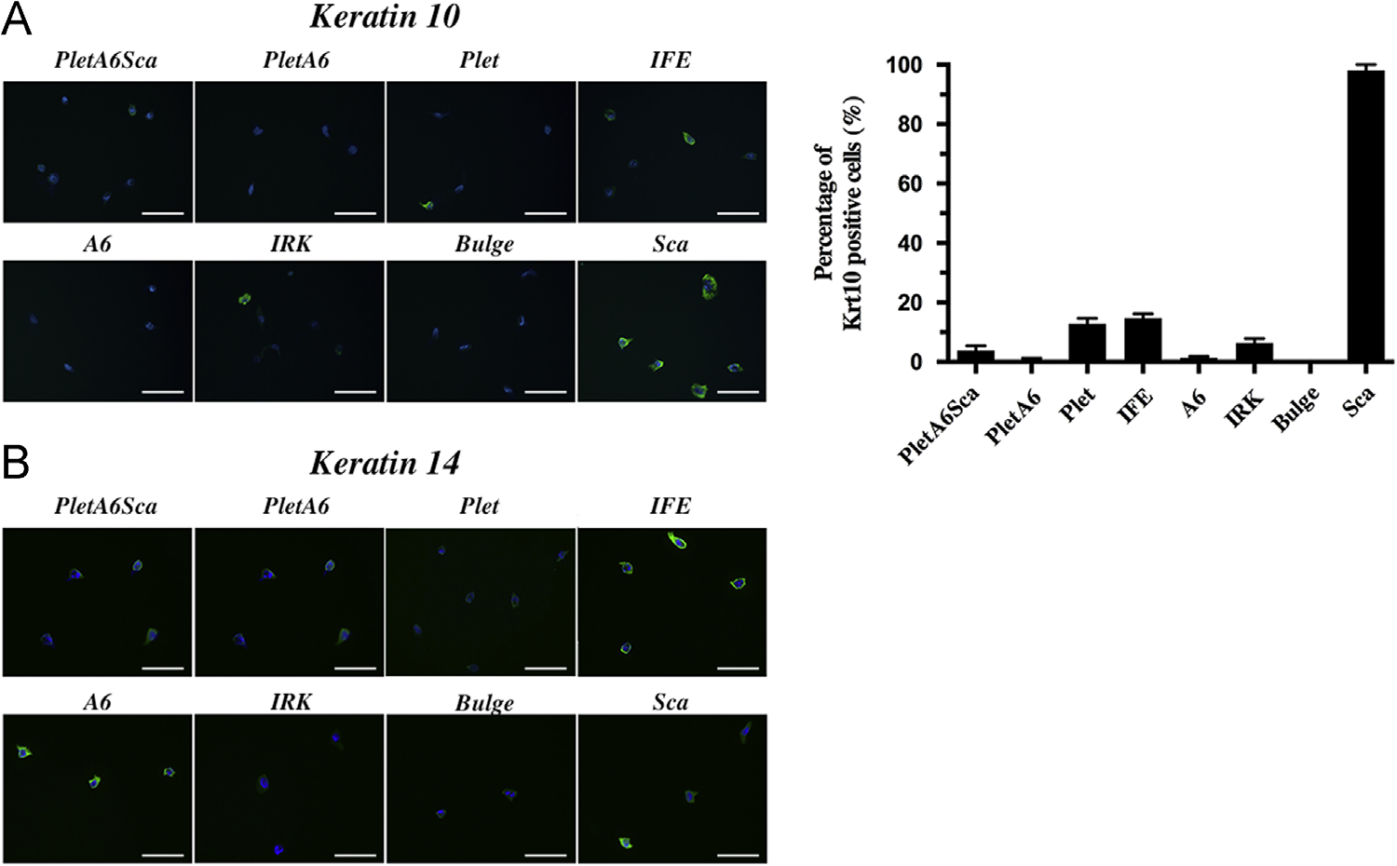
Krt10 and Krt14 immunofluorescence staining of the different epidermal populations. Freshly isolated keratinocytes were stained with antibodies against Itgα6, CD34, Sca-1 and Plet-1. Using flow cytometry, the different keratinocyte populations *PletA6Sca*, *PletA6*, *Plet*, *IFE*, *A6*, *IRK*, *Bulge* and *Sca* were sorted on slides and cytospun at 200g for 5 min. Cells were fixated and stained with either anti-Krt10 (A) or anti-Krt14 (B) primary antibodies with Alexa Fluor 488-conjugated secondary antibody. Using fluorescence microscopy, the numbers of green positive cells were visualized for each population. (A) *Sca* shows almost 100% of Krt10 positive cells, while *IFE* and *Plet* approximately consist of 15–20% Krt10-expressing cells. A summary graph of Krt10 positive cells is shown to the right. Error bars represent SEM (*n*=3). (B) With all keratinocytes having levels of the Krt14 protein, *IFE* and *A6* show the highest Krt14-intensities of all the populations. Scale bar: 50 μm.

**Fig. 5:**
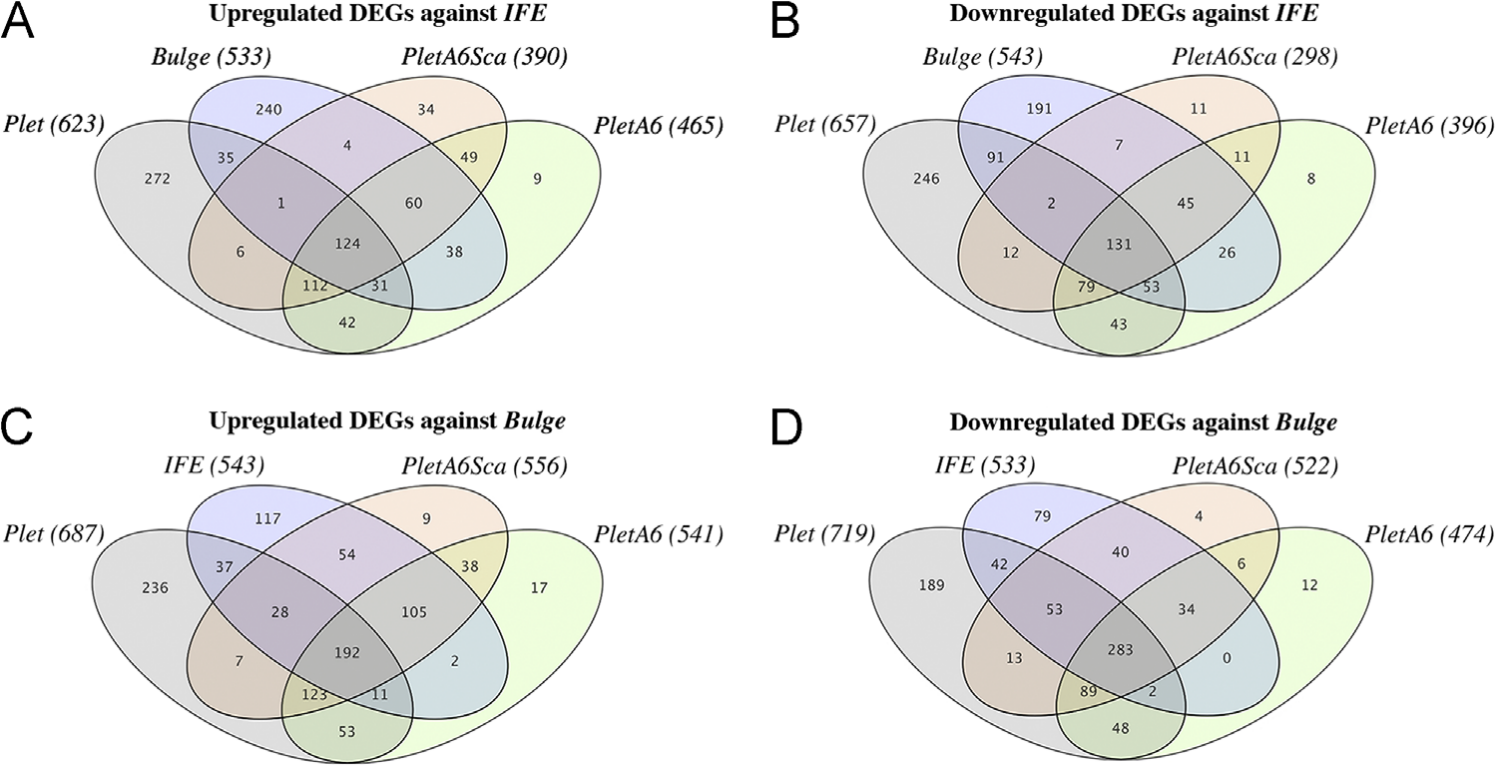
Venn-diagrams show the gene expression similarities between the different populations. After generating gene expression profiling for each of the populations on microarray, the data was normalized and filtered on present probes. By using ANOVA analysis with Benjamini–Hochberg false discovery rate (*p* adj.<0.05), the populations were compared pair-wise to reveal the number of upregulated- and downregulated differently expressed genes (DEGs) (logFC>0.5). Subsequently, the gene expression similarities between two or several populations can be visualized by using Venn-diagrams. The number of commonly differently expressed genes towards a reference population can be found in each of the areas covered by the population-specific ellipses. (A) Upregulated DEGs against *IFE* reference. (B) Downregulated DEGs against *IFE* reference. (C) Upregulated DEGs against *Bulge* reference. (D) Downregulated DEGs against *Bulge* reference. For each population, the total numbers of differently expressed genes against the reference population are written in parenthesis. *PletA6Sca* and *PletA6* show high similarity by sharing most of their differently expressed genes with each other and the two remaining populations, regardless of reference. On the other hand, *Plet* and *Bulge* display greater distinctiveness with over a third of their total differently expressed genes not being shared by any of the other populations.

**Fig. 6:**
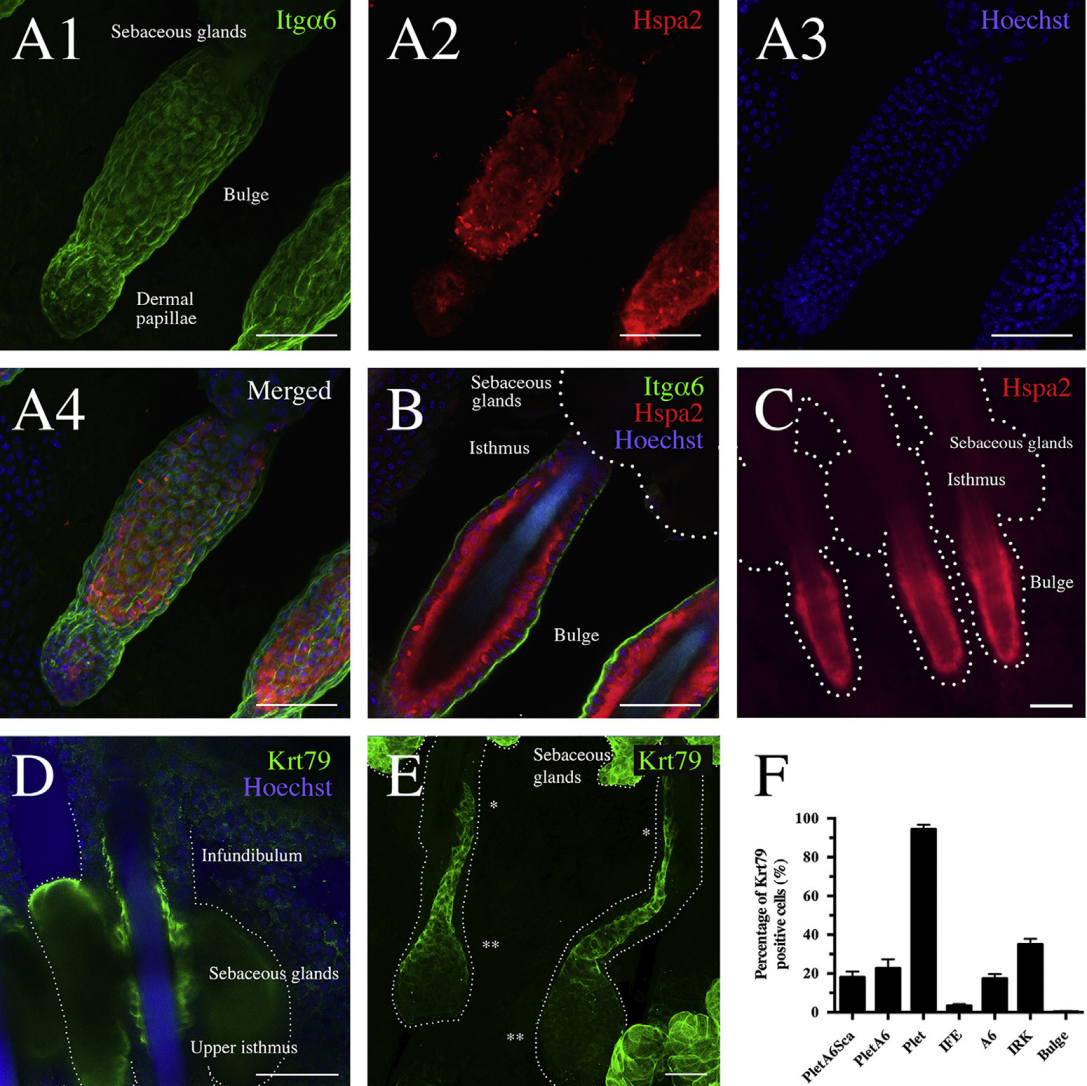
Whole-mount immunofluorescence staining of Itgα6, Hspa2 and Krt79. (A1–A4) Stacked confocal sections of murine whole-mounts stained with antibodies targeting (A1) Itgα6 (green) and (A2) Hspa2 (red). (A3) Hoechst nucleus staining (blue). (A4) Merged images. (B) Confocal section view of stained whole-mounts with Itgα6 (green), Hspa2 (red) and Hoechst (blue). Dotted line marks sebaceous glands. (C) Immunofluorescence microscopy of Hspa2-stained whole-mounts (red). Dotted lines mark hair follicle outer layer. (D–E) Confocal sections of whole-mounts stained with Krt79 antibody (green). Dotted lines mark hair follicle outer layer. (D) Krt79 (green) is expressed by the inner root sheath keratinocytes within upper isthmus and infundibulum. Nucleuses are stained with Hoechst (blue). (E) Krt79 expression (green) in two hair follicle formations during the anagen phase. Single asterisk denote old hair follicles and double asterisk marks the new channels. (F) Summarizing the percentages of Krt79-expressing keratinocytes within the different populations. Directly isolated murine dorsal keratinocytes were stained with antibodies against Plet-1, Sca-1, Itgα6 and CD34 to isolate the populations *PletA6Sca*, *PletA6*, *Plet*, *IFE*, *A6*, *IRK* and *Bulge*. By using flow cytometry, the populations were sorted onto slides and fixated before further staining with anti-Krt79 antibody and visualized using fluorescence microscopy. For each population, the percentages of Krt79-positive cells were thereafter calculated. Scale bar: 50 μm. (For interpretation of the references to color in this figure legend, the reader is referred to the web version of this article).

Dorsal keratinocytes were isolated from female C57Bl/6 mice in the age of 7–9 weeks and sorted using flow cytometry. Cells were stained with conjugated antibodies and visualized by fluorescence microscopy. Global microarray data was used to generate Venn diagrams, which show the number of shared differently expressed genes (DEGs) between the different populations and a reference. See Fig. 1, Fig. 2, Fig. 3, Fig. 4, Fig. 5, Fig. 6.

## 2 Experimental design, materials and methods

### 2.1 Isolation of murine dorsal keratinocytes

Female 7–9 weeks old C57Bl/6 mice were sacrificed by cervical dislocation. Their backs were shaved using razor machine and the dorsal skin peeled off with sterile forceps and scissors. The dermal fat was scraped away using sterile scalpel and the skin was disinfected in 1% Betadine for 30 s, 70% EtOH for 30 s and washed in PBS. With the epidermal side facing upwards, the skins were kept floating on 0,25% trypsin supplemented with 5 mM EDTA over night at 4 °C. Following day, the epidermal cells were gently scraped away from dermis using sterile scalpels into chilled DMEM with 10% FCS and 100x penicillin-streptomycin, centrifuged at 200 g, 4 °C for 10 min and washed in PBS supplemented with 0.1% Bovine Serum Albumin.

### 2.2 Flow cytometry and immunofluorescence staining

To isolate the different populations using flow cytometry, freshly isolated keratinocyte cell suspension was stained for 30 min on ice with surface antibodies Brilliant Violet 421-conjugated CD34 (clone RAM34; BD Biosciences); PE-conjugated Itgα6 (CD49f, clone GoH3; BD Biosciences); PE-Cy/7-conjugated Sca-1 (Ly-6A/E, clone D4; BD Biosciences); APC-conjugated Plet-1 (clone 33A10; Mubio) using Lighting-link labeling kit (Innova Biosciences). This panel of four antibodies has been shown to increase the number of populations within the murine hair follicle and in particular increase the resolution of the pilosebaceous region by flow cytometry [1]. To ensure that our multipanel of antibody-fluorochromes does not generate false positive cells from spectral overlap, we conducted a fluorochrome minus one (FMO) assay. In essence, by staining the keratinocyte suspension with all antibodies except one, any false emission signal for that fluorochrome will be revealed. This assay of antibody-exclusion was made for all antibodies in separate tubes (Fig. 1).

Using FACSAria III cell sorter (405 nm violet, 488 nm blue, 561 nm yellow-green and 633 nm red lasers), keratinocytes were first gated on viability (Fig. 2**A**) and small size (Fig. 2**B**) after which the different epidermal populations were identified and sorted on slides or into tubes with chilled lysis-buffer. Large cells of the sebaceous glands with intermediate levels of Sca-1 were avoided due to the size-exclusion (Fig. 2**C–E**). In order to acquire single cell high-magnification images of from each population, freshly isolated dorsal keratinocytes stained against Sca-1, CD34, Itgα6 and Plet-1 were analyzed though a ImageStream flow cytometer (Fig. 3). After sorting the populations onto glass slides, the cells were fixated for 30 s in methanol and stained with anti-Keratin 10 or anti-Keratin 14 primary antibodies with secondary antibody Alexa Fluor 488 and visualized using fluorescence microscopy (Leica Letiz DMRB) (Fig. 4**A and B**).

### 2.3 Venn diagrams and the number of shared differently expressed genes (DEGs)

After sorting each of the populations into separate tubes containing lysis-buffer, their total RNA were isolated using Maxwell 16 LEV simplyRNA kit (Promega) and profiled on MouseWG-6 v2 BeadChips (Illumina). By generating Venn diagrams, the numbers of upregulated- and downregulated differently expressed genes (DEGs) were visualized between one reference population and the four other populations (Fig. 5**A–D**).

### 2.4 Whole-mount preparation and visualization of Hspa2- and Krt79-expression

Murine tail skin was isolated and peeled of the bone after performing a longitudinal section using a scalpel. The skin was cut into 0.5×0.5 cm^2^ pieces and kept in PBS with 5 mM EDTA at 37 °C for 5 h after which whole-mount epidermal sheets were gently separated from dermis using forceps. The pieces were fixated in 2% neutral buffered formaldehyde Cellpath Ltd) for 10 min and kept in PBS at 4 °C until further processing. The sheets were stained with gentle movement for 1 h using primary antibodies PE-conjugated anti-Itgα6 (CD49f, clone GoH3, and BD Biosciences), anti-Hspa2 (Clone EPR4596; Abcam), anti-Keratin 79 (clone Y-17; Santa Cruz biotechnology) and secondary Alexa Fluor 488 antibody. Images were acquired using fluorescence microscope (Leica Letiz DMRB) or LSM 710 confocal microscope (405 nm, 488 nm, and 633 nm lasers; Carl Zeiss) (Fig. 6**A1**–**F**).

## References

[1] Gunnarsson A.P., Christensen R., Li J., Jensen U.B. (2016). Global gene expression and comparison between multiple populations in the mouse epidermis. Stem Cell Res..

[2] Jones P.H., Harper S., Watt F.M. (1995). Stem cell patterning and fate in human epidermis. Cell.

[3] Jensen U.B. (2008). A distinct population of clonogenic and multipotent murine follicular keratinocytes residing in the upper isthmus. J. Cell Sci..

